# Asgard archaeal selenoproteome reveals a roadmap for the archaea-to-eukaryote transition of selenocysteine incorporation machinery

**DOI:** 10.1093/ismejo/wrae111

**Published:** 2024-06-19

**Authors:** Biyan Huang, Yao Xiao, Yan Zhang

**Affiliations:** Shenzhen Key Laboratory of Marine Bioresources and Ecology, Brain Disease and Big Data Research Institute, College of Life Sciences and Oceanography, Shenzhen University, Shenzhen 518055, Guangdong Province, P. R. China; Shenzhen Key Laboratory of Marine Bioresources and Ecology, Brain Disease and Big Data Research Institute, College of Life Sciences and Oceanography, Shenzhen University, Shenzhen 518055, Guangdong Province, P. R. China; Shenzhen Key Laboratory of Marine Bioresources and Ecology, Brain Disease and Big Data Research Institute, College of Life Sciences and Oceanography, Shenzhen University, Shenzhen 518055, Guangdong Province, P. R. China; Shenzhen-Hong Kong Institute of Brain Science-Shenzhen Fundamental Research Institutions, Shenzhen 518055, Guangdong Province, P. R. China

**Keywords:** selenium, selenocysteine, selenoprotein, secis element, asgard archaea, evolution, comparative genomics

## Abstract

Selenocysteine (Sec) is encoded by the UGA codon that normally functions as a stop signal and is specifically incorporated into selenoproteins via a unique recoding mechanism. The translational recoding of UGA as Sec is directed by an unusual RNA structure, the SECIS element. Although archaea and eukaryotes adopt similar Sec encoding machinery, the SECIS elements have no similarities to each other with regard to sequence and structure. We analyzed >400 Asgard archaeal genomes to examine the occurrence of both Sec encoding system and selenoproteins in this archaeal superphylum, the closest prokaryotic relatives of eukaryotes. A comprehensive map of Sec utilization trait has been generated, providing the most detailed understanding of the use of this nonstandard amino acid in Asgard archaea so far. By characterizing the selenoproteomes of all organisms, several selenoprotein-rich phyla and species were identified. Most Asgard archaeal selenoprotein genes possess eukaryotic SECIS-like structures with varying degrees of diversity. Moreover, euryarchaeal SECIS elements might originate from Asgard archaeal SECIS elements via lateral gene transfer, indicating a complex and dynamic scenario of the evolution of SECIS element within archaea. Finally, a roadmap for the transition of eukaryotic SECIS elements from archaea was proposed, and selenophosphate synthetase may serve as a potential intermediate for the generation of ancestral eukaryotic SECIS element. Our results offer new insights into a deeper understanding of the evolution of Sec insertion machinery.

## Introduction

Selenium (Se) is an essential trace element for many organisms, including prokaryotes, protists, animals, and humans [1, 2]. It mainly occurs in the form of selenocysteine (Sec, the 21st amino acid), which is cotranslationally incorporated into selenoproteins by recoding the opal (UGA) codon [3]. These proteins play critical roles in redox homeostasis, immune responses, antiviral defense, hormone metabolism, and several other important cellular processes [4–6]. To date, dozens of selenoprotein families have been reported in both prokaryotes and eukaryotes, many of which have the functions of antioxidation and detoxification [7–9].

The biosynthesis of Sec and its insertion into proteins require complex molecular machinery that has generally been elucidated in all three domains of life [10–13]. In bacteria, recognition of UGA codon as a Sec site requires a *cis*-acting stem-loop structure, designated Sec insertion sequence (SECIS) element, within the selenoprotein mRNA immediately downstream of the Sec-encoding UGA codon and several *trans*-acting factors including Sec synthase (SelA), Sec-specific elongation factor (SelB), tRNA^[Ser]Sec^, and selenophosphate synthetase (SelD, or named SEPHS2 in eukaryotes). In archaea and eukaryotes, somewhat different steps and enzymes, such as archaeal/eukaryotic Sec synthase (SecS), archaeal/eukaryotic Sec-specific elongation factor EFSec (homolog of SelB with conserved N-terminal region but different C-terminal parts among bacteria, archaea, and eukaryotes), and *O*-phosphoseryl-tRNA^Sec^ kinase (PSTK), are needed for the incorporation of Sec into selenoproteins. The SECIS elements in archaea and eukaryotes are both located in the 3′-untranslated regions (3’-UTRs) of selenoprotein genes instead of the coding region. However, the absence of several eukaryotic proteins (such as SECIS binding protein 2 (SBP2) and tRNA selenocysteine 1 associated protein 1) in archaea highlights the difference in Sec incorporation between the two domains of life [14]. A general scheme of Sec biosynthesis and its insertion into proteins in archaea is shown in, See online supplementary material for a colour version of this, Fig. S1.

It has been proposed that Sec utilization is an ancient trait derived from the last universal common ancestor and was once common to almost all species in bacteria and eukaryotes but has been lost in many lineages independently [15–17]. Although the Sec encoding system in eukaryotes is generally thought to originate from within archaea, there are obvious distinctions between them, in particular, the lack of significant resemblance in sequence or structure of their SECIS elements [17–19]. The eukaryotic SECIS (eSECIS) elements consist of two stems separated by an internal loop, a SECIS core structure with an unusual 5′-GA_GA-3′ non-Watson-Crick base pair quartet (a typical kink-turn motif) located at the base of the upper stem (preceding the first GA is always AU (rarely GU) to form the AUGA tetranucleotide motif), and an apical loop (mostly containing two or three unpaired adenines). The archaeal SECIS elements previously reported in *Euryarchaeota* (named eaSECIS thereafter) differ from those in eukaryotes and possess two stems separated by an internal bulge with a conserved 5′-GAA_A-3′ motif, three consecutive C/G-G/C (or S-S, S represents C or G) pairs in the upper stem, and an apical loop with variable length. In the last decade, computational studies have been carried out to explore the distribution of the Sec encoding trait and selenoproteome (the whole set of selenoproteins in a Sec-utilizing organism) in a variety of sequenced eukaryotes (such as vertebrates, algae, and fungi), which provide clues for a better understanding of the dynamic evolution of Sec utilization in different taxa of eukaryotes [20–24]. In contrast, Sec usage in archaea is quite restricted and was only detected in two lineages of the *Methanomada* group from the *Euryarchaeota* phylum, *Methanococcales* and *Methanopyrales* (*Methanopyrus*), which contain a limited number of selenoproteins [9, 25, 26].

In recent years, a novel archaeal superphylum, the Asgard archaea, has been identified, which includes *Lokiarchaeia*, *Thorarchaeia*, *Heimdallarchaeia*, *Odinarchaeia*, and several other clades [27–30]. This deep-branching monophyletic group of archaea appears to harbor numerous eukaryotic-like features and have been considered as the closest archaeal relatives of eukaryotes [31]. Previous studies have reported that several *Lokiarchaeia*, *Thorarchaeia*, and *Jordarchaeia* species have both Sec encoding machinery and conserved RNA structures that are similar to eSECIS elements, which suggest the existence of intermediate forms between archaeal and eukaryotic Sec insertion systems [32–34]. So far, however, the presence of the Sec utilization trait in many other phyla of Asgard archaea and details for the transition of Sec machinery from archaea to eukaryotes remain a mystery.

In this study, we utilized >400 Asgard archaeal genomes reconstructed from different metagenomic projects to investigate Sec utilization in this superphylum. We analyzed the distribution of key components involved in the Sec pathway and generated a more comprehensive picture of Sec utilization in Asgard archaea. Identification of the selenoproteomes shows the presence of additional selenoprotein families in different taxa of Asgard archaea. Further analysis of possible SECIS structures in Asgard archaeal and euryarchaeal selenoprotein genes suggests a wider and earlier presence of key features of eSECIS elements across archaeal lineages. These data provide new evidence for the origin and related evolutionary processes of eukaryotic Sec incorporation machinery.

## Materials and methods

### Genomic data acquisition, phylogenomic inference, and taxonomic assignment

A total of 409 metagenome-assembled genomes (MAGs) of Asgard archaea were downloaded from NCBI Genbank database (as of November 2022), which have estimated average completeness and redundancy values of 76.6% and 3.22%, respectively. Genomic sequence alignment, phylogenomic inference, and taxonomic rank assignment were performed by using the Genome Taxonomy Database (GTDB) toolkit GTDB-Tk (version 2.3.2) [35]. The evolutionary relationship of different Asgard archaea was inferred via maximum-likelihood trees based on 53 archaeal single-copy marker proteins provided by GTDB-Tk. All Asgard archaea examined in this study were finally divided into nine normalized taxonomic ranks/clades: *Baldrarchaeia*, *Heimdallarchaeia/Hodarchaeales*, *Hermodarchaeia*, *Jordarchaeia*, *Lokiarchaeia/Helarchaeales*, *Odinarchaeia*, *Sifarchaeia/Borrarchaeales*, *Thorarchaeia*, and *Wukongarchaeia*.

### Identification of Sec encoding system and selenoprotein genes in Asgard archaeal genomes

We used *Methanocaldococcus jannaschii* SelD, SecS, EFSec, and PSTK sequences (Table S1) as queries to search for components of the Sec encoding system. The tblastn program was used to identify a primary set of genes encoding homologs with a cutoff e-value of 0.01 and at least 25% coverage of the alignment region in query or subject sequences. Additional iterations of tblastn searches were then performed within each clade (phylum or class) to identify additional homologous sequences using selected sequences from the same or closely related clades in the primary data set. In addition, the iterative profile hidden Markov model (profile-HMM) search method, JackHMMER, was used to search against the genomic sequences for distant homologs that match the HMMs of corresponding proteins with e-value <0.05 [36]. All obtained sequences were submitted to the NCBI-CDD web server to verify orthologous genes by analyzing their conserved domain information (such as COG, Pfam, and TIGRFAMs) [37–39]. If no appropriate protein domain could be assigned, an extended all-versus-all sequence similarity search procedure between different species was implemented to define orthologs based on the bidirectional best hit approach using blastp and/or tblastx programs with the threshold e-value of 0.001 [40, 41]. Because SelD is a selenoprotein in many organisms, the presence of SelD in Asgard archaea was further verified using the strategy for the identification of selenoprotein genes (see below). The tRNA^[Ser]Sec^ was predicted using Secmarker (version 0.4) and tRNAScan-SE (version 2.0.9) tools with default settings [42, 43].

We also collected representative sequences for all previously reported prokaryotic and eukaryotic selenoprotein families (Table S1) [9, 17, 24, 44, 45]. These sequences were used to search against the Asgard archaeal genomic sequences for selenoprotein homologs via tblastn with default parameters. Distant homologs were further identified by using additional iterations of tblastn searches. The tblastn output for each selenoprotein sequence was parsed, and only the Sec/TGA pairs (Sec in a query sequence was aligned with TGA in the nucleotide sequence from the target genomic dataset) were selected. A reasonable open reading frame (ORF) was predicted for each TGA-containing nucleotide sequence using the OrfFinder program [46]. Similarly, the JackHMMER program was also used for the verification of distant homologs of each selenoprotein family based on a self-built TGA-containing ORF dataset derived from Asgard archaeal MAGs (each TGA in the genome was considered as a potential Sec-encoding codon and extended upstream and downstream to obtain a relevant ORF sequence). Redundant selenoprotein sequences were removed, and the presence of cysteine(Cys)-containing homologs (in which Sec is replaced by Cys) was analyzed via blastp search against the NCBI non-redundant protein database (with default parameters) to ensure the accuracy of selenoprotein genes. For each selenoprotein family, multiple sequence alignment (see below for details) was also carried out to verify the conservation of Sec and its flanking regions in selenoprotein sequences identified here. All Sec machinery genes, selenoprotein genes, and genomic regions of 5 kb upstream and downstream of these genes were extracted and further analyzed to remove potential contaminations derived from unrelated organisms (such as bacteria) using blastn and blastx programs with default parameters.

As almost all Asgard archaeal genomes were not fully sequenced and might contain multiple strains, the Sec utilization trait was finally verified by the requirement for (i) the presence of at least three genes of the Sec encoding system (including tRNA^[Ser]Sec^) and (ii) the presence of at least one known selenoprotein gene.

### Development of different models of Asgard archaeal SECIS elements

**Figure 1:**
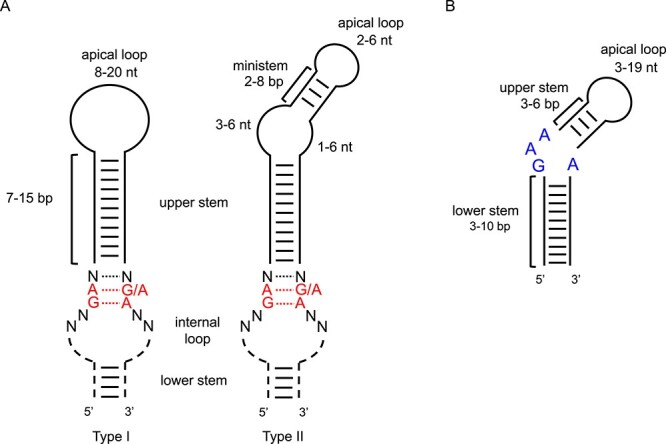
**Consensus structural models of Asgard archaeal SECIS elements.** (A) Two types of the eSECIS-like model; (B) the eaSECIS-like model. The allowed lengths of different stems and loops are indicated. Conserved nucleotides are shown in different colors, including the non-Watson-crick base pair quartet (eSECIS core) and the GAA_A bulge (eaSECIS core) in different models. N represents any nucleotide.

As several eSECIS-like structures have been previously reported in *Lokiarchaeia*, *Thorarchaeia*, and *Jordarchaeia* [32–34], they could serve as good references for the construction of a preliminary model for Asgard archaeal SECIS elements. Considering that eSECIS elements fall into two basic classes (types I and II) [47] and that some SECIS elements in other Asgard archaeal phyla might have distinct characteristics different from those in *Loki-/Thorarchaeia*, we developed a general model with looser criteria for the key regions of eSECIS-like structures (SECIS quartet core + upper stem + apical loop) in Asgard archaea (Fig. 1A): (i) type I eSECIS-like model (without ministem in the apical loop): the nucleotides within the SECIS core are 5′-GA_[G/A]A-3′, the length of the upper stem is 7–15 bp (number of mismatches ≤2), and the length of the apical loop is 8–20 nt; (ii) type II eSECIS-like model (with ministem in the apical loop): the nucleotides within the SECIS core are 5′-GA_[G/A]A-3′, the length of the upper stem is 7–15 bp (number of mismatches ≤2), the length of the ministem is 2–8 bp (number of mismatches ≤1), the bulge between the upper stem and the ministem consists of 4–12 nt, and the length of the apical loop is 2–6 nt.

We also developed a eaSECIS-like structural model based on the following constraints: the length of the lower stem is 3–10 bp, the length of the upper stem is 3–6 bp, a pronounced 5′-GAA_A-3′ bulge is present between stems, and the length of the apical loop is 3–19 nt (Fig. 1B).

### Prediction of SECIS-like structures in Asgard archaeal selenoprotein genes

It has been reported that the distance between the canonical eaSECIS element and the 3′ end of selenoprotein genes in *M. jannaschii* is generally short [18]. Moreover, the UGA stop codons of selenoprotein genes in *Lokiarchaeia* often overlap with the AUGA motif in their eSECIS-like structures [32]. Thus, we retrieved the genomic regions 30 nt upstream and 600 nt downstream of the 3′ end of selenoprotein genes identified here. We used the SECISearch3 tool [48] to search for possible eSECIS-like structures with different parameters, but no significant results could be obtained. We then used the PatScan tool [49] to search for fragments satisfying the pattern 5’-NNGAN(22–65 nt)[G/A]ANN-3′, which was designed to fit the constraints of the key regions of both type I and type II eSECIS-like models (type I pattern: r1={au,ua,gc,cg,gu,ug} NNGAN p1=7...15 8...20 r1~p1[2,1,1] (NGANN | NAANN); type II pattern: r1={au,ua,gc,cg,gu,ug} NNGAN p1=6...14 3...6 p2=2...8 2...6 r1~p2[1,1,1] 1...6 r1~p1[2,1,1] (NGANN | NAANN)). Genomic sequences that meet the criterion were collected for secondary structure prediction using Mfold (version 3.6) or RNAfold (version 2.6.4) programs with default parameters [50, 51]. Predicted RNA structures were analyzed against the eSECIS-like structural models and free energy criteria (< −5.0 kcal/mol threshold previously used for the analysis of SECIS candidates in *Lokiarchaeia* [32]). Sequences satisfying the constraints for these models were remained for further analysis.

Prediction of eaSECIS-like structures was performed using the same procedure with different constraints defined in the eaSECIS-like model (pattern for PatScan search: r1={au,ua,gc,cg,gu,ug} p1=3...10 GAA p2=3...6 3...19 r1~p2[0,1,1] A r1~p1[1,1,1]). Finally, all selenoprotein genes with eSECIS- and/or eaSECIS-like structures were obtained.

### Identification of selenoprotein genes and eaSECIS elements in euryarchaeal genomes

We downloaded the genomic sequences of ~30 organisms belonging to *Methanococcales* and *Methanopyrus* from NCBI, which are the only known archaea (except Asgard archaea) that use Sec thus far [9, 25]. All known selenoprotein sequences in *M. jannaschii* (Table S2) were used as seeds to identify selenoprotein genes in other organisms with the same procedure described above. Canonical eaSECIS elements in these selenoprotein genes were further identified using the same strategy discussed previously [44].

### Multiple alignment and phylogenetic analysis

Multiple sequence alignment for selenoproteins was conducted using MAFFT (version 7.505) with the options “--anysymbol --ep 0 --genafpair --maxiterate 1000” [52]. Sequences shorter than 35 aa were excluded from further phylogenetic analysis. For the phylogeny of each selenoprotein family, all selenoprotein sequences detected in Asgard archaea, selenoprotein sequences found in *Methanococcales* and *Methanopyrus* species (if any), and representative Cys-containing homologs chosen from the top blast hits (using NCBI online blastp with e-value <0.001) from Asgard archaea and *Euryarchaeota* were aligned using MAFFT with the options “--anysymbol --ep 0 --genafpair --maxiterate 1000”. The alignment result was modified to trim off poorly aligned regions using trimAl software (version 1.4) with the “-automated1” option [53]. A maximum-likelihood phylogenetic tree was generated from the alignment using IQ-TREE software (version 1.6.12) with the best-fit protein model as determined by ModelFinder and 1000 ultrafast bootstrap pseudoreplicates (parameters: -m MFP -bb 1000 -bnni -alrt 1000 -nt AUTO) [54]. For the phylogenetic analyses of genes surrounding selenoprotein genes and Sec machinery genes, homologous sequences of their proteins from diverse archaeal supergroups (Asgard archaea, *Euryarchaeota*, TACK, and DPANN groups) were initially collected using NCBI online blastp program (e-value <0.001), and orthologous sequences were identified using the same strategy described above. Representative sequences covering the entire archaeal taxonomic diversity were then selected from different clades of these archaeal supergroups. These sequences were aligned using MAFFT with the options “--localpair --maxiterate 1000”, and the alignment was trimmed using trimAl with the “-automated1” option. The phylogenetic trees were constructed using IQ-TREE with the LG+C60+F+R model, which was the best-suited model for most selenoproteins examined here (parameters: -m LG+C60+F+R -bb 1000 -alrt 1000 -nt AUTO). Paralogous sequences were used as outgroups to root these trees. All phylogenetic trees were further visualized using the Interactive Tree Of Life online tool (version 6.3) [55].

Multiple alignments of nucleotide sequences surrounding the key regions of eSECIS-like structures in Asgard archaea and EFSec proteins in archaea were performed using MAFFT with default settings.

## Results

### Occurrence of the sec utilization trait in Asgard archaea

**Figure 2:**
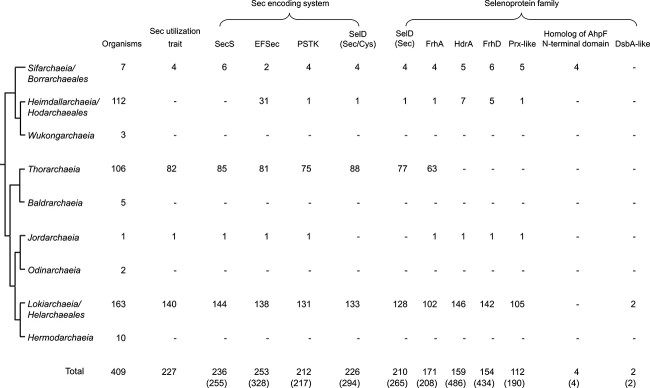
**Distribution of components of the sec encoding system and selenoprotein families in Asgard archaea.** The phylogenetic tree constructed on the basis of 53 archaeal-specific marker proteins in GTDB is simplified to only show major clades of Asgard archaea. The number of organisms containing corresponding Sec encoding component and selenoprotein family is shown. The total number of sequences for each protein family is shown in parenthesis. SecS, archaeal/eukaryotic Sec synthase; EFSec, archaeal/eukaryotic Sec-specific elongation factor; PSTK, *O*-phosphoseryl-tRNA^Sec^ kinase; SelD, selenophosphate synthetase; FrhA, coenzyme F_420_−/methylviologen-reducing hydrogenase alpha subunit; HdrA, heterodisulfide reductase subunit A; FrhD, coenzyme F_420_-reducing hydrogenase delta subunit; Prx-like, peroxiredoxin-like protein; DsbA-like, disulfide bond formation protein A-like protein.

We examined the distribution of Sec encoding system in >400 Asgard archaeal genomes, which has demonstrated the largest picture for the use of this rare amino acid in the newly identified archaeal superphylum thus far (Fig. 2, details in Table S3). We also checked for the occurrence of SBP2, an essential component during Sec insertion in eukaryotes, which interacts with the conserved GA_GA quartet within the eSECIS element. No *sbp2* gene could be detected in any Asgard archaeal genomic sequences. This observation is consistent with previous findings that SBP2 is not needed for SECIS recognition in *Euryarchaeota* and several Asgard archaea such as *Lokiarchaeia* and *Sifarchaeia* [25, 32, 34].

Based on the criteria for Sec utilization described in this study, a total of 227 organisms (55.5% of sequenced Asgard archaea) belonging to four subdivisions were found to have the Sec utilization trait. Particularly, the majority of sequenced organisms in *Lokiarchaeia/Helarchaeales* (85.9%), *Thorarchaeia* (77.4%), and *Sifarchaeia/Borrarchaeales* (57.1%) utilize Sec. Other clades appear to lack Sec usage. We also identified Sec-containing homologs of known selenoprotein families in a small number of organisms that lack all or most components of the Sec encoding system, e.g. several species belonging to *Heimdallarchaeia/Hodarchaeales*. Considering that only a very limited number of genomes are available for most Asgard clades and that almost all Asgard archaeal genomes are still incomplete, the possibility either that additional Sec-utilizing organisms are present or that genes involved in the Sec encoding system and/or known selenoprotein genes have not been sequenced could not be excluded. Nevertheless, the widespread occurrence of the Sec utilization trait in Asgard archaea implies that it is an essential trait for the ancestor of these organisms. However, several lineages might have lost the ability to use Sec. In the following part, we mainly focus on organisms containing the Sec utilization trait for better understanding the diversity and evolutionary trends of selenoproteins in Asgard archaea.

We also examined the occurrence of other known Se utilization pathways, including 2-selenouridine (a modified base present at the wobble position of some prokaryotic tRNAs), Se-containing cofactor (used by certain molybdenum-containing enzymes in prokaryotes), and the recently identified selenoneine (a Se analog of ergothioneine in prokaryotes) [56–58]. None of them could be detected, suggesting that Sec is the only biological form of Se in Asgard archaea.

### Distribution of selenoproteins and selenoproteome in Asgard archaea

By collecting representative sequences for previously reported selenoprotein families and searching for appropriate Sec/TGA pairs, we identified 1439 selenoprotein sequences in Sec-utilizing Asgard archaea, which belonged to seven selenoprotein families (Fig. 2, details in Table S3). Among them, five selenoprotein families are widespread and have been previously reported in *Lokiarchaeia* [32], including SelD, coenzyme F_420_−/methylviologen-reducing hydrogenase alpha subunit (FrhA, MvhA, or VhuU), heterodisulfide reductase subunit A (HdrA), coenzyme F_420_-reducing hydrogenase delta subunit (FrhD, MvhD, or VhuD), and peroxiredoxin(Prx)-like protein (detected in 87.2%, 68.3%, 58.1%, 57.3%, and 43.2% of Sec-utilizing Asgard archaea, respectively), accounting for 99.6% of all selenoprotein sequences (scatter plots displaying the e-value and coverage values for homology search for members of these selenoprotein families in Asgard archaea are provided in, See online supplementary material for a colour version of this, Fig. S2). For these selenoprotein families, phylogenetic analyses of all selenoprotein sequences detected here and additional Sec-/Cys-containing homologs from other archaea further confirm that these selenoproteins are derived from Asgard archaea (See online supplementary material for a colour version of this Figs S3-S7). SelD was the most widely used selenoprotein that was found in >90% of Sec-utilizing Asgard archaea. A number of *Loki-/Thorarchaeia* species (51 organisms) possess two or more *selD* genes, which was rarely observed in other prokaryotic organisms. This suggests that Sec-containing SelD might either play a more active and important role in Sec biosynthesis or have acquired new function in these organisms. Although numbers of organisms containing *hdrA* and/or *frhD* genes were less than those containing *selD* and/or *frhA*, most of them have multiple members of the two families, resulting in the fact that HdrA and FrhD have the most abundant selenoprotein sequences among all detected selenoprotein families in Asgard archaea. We found that *hdrA* and *frhD* genes are always located together with very short intergenic regions on the same strand in various Asgard archaeal MAGs. Such a *hdrA*-*frhD* gene cluster was also conserved in some other archaea such as *Methanococcales* species, which may contribute to simultaneous Sec incorporation into both selenoproteins by using the very same machinery (say, same SECIS element).

Several other prokaryotic selenoproteins were also found in certain Asgard species, including homolog of AhpF N-terminal domain (four sequences in *Sifarchaeia/Borrarchaeales*) and disulfide bond formation protein A(DsbA)-like protein (two sequences in *Lokiarchaeia/Helarchaeales*), which have not been reported in archaea (Fig. 2). Further phylogenetic analysis of the origin of these selenoprotein sequences reveals that they might have newly evolved from their Cys-containing homologs (See online supplementary material for a colour version of Figs S8 and S9). In contrast, no eukaryotic-specific selenoprotein family could be detected in Asgard archaea.

**Figure 3:**
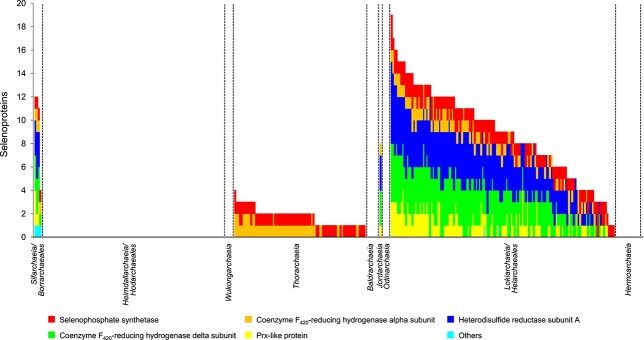
**Distribution of selenoproteomes in Asgard archaea.** Individual selenoprotein families are indicated with different colors of blocks.

We further characterized the selenoproteomes encoded in Asgard archaeal genomes (Fig. 3 and Table S3). In general, organisms belonging to *Sifarchaeia/Borrarchaeales* and *Lokiarchaeia/Helarchaeales* appeared to have larger selenoproteomes than others. Considering that all Asgard archaeal genomes analyzed here were derived from metagenomic projects, it is possible that multiple closely related species or strains are mixed in the same MAG. Further efforts are expected to obtain more accurate genomic sequences for Asgard archaea.

### Investigation of SECIS elements in Asgard archaea

Aiming to identify potential SECIS elements in the 3’-UTRs of Asgard archaeal selenoprotein genes, we developed general models for the key regions of eSECIS- and eaSECIS-like structures. The majority of selenoprotein genes (1224 sequences, 85.1%) have eSECIS-like structures. We mainly focused on the five widespread selenoprotein families (SelD, FrhA, HdrA, FrhD, and Prx-like protein) to explore conserved features of these structures (Table S4). Because *hdrA* and *frhD* are always neighbor genes in Asgard archaeal genomes, almost all of them share the same eSECIS-like structures.

**Figure 4:**
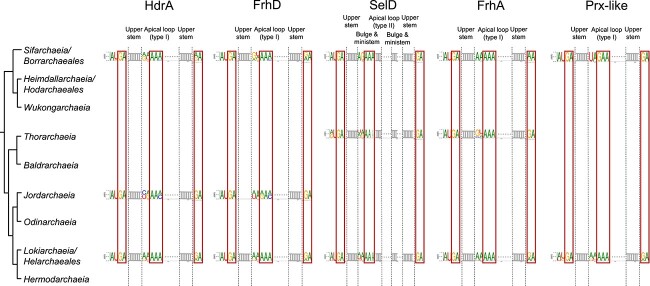
**Sequence logos of key regions of eSECIS-like structures (major forms) in different selenoprotein genes.** The logo map was generated using WebLogo (version 3.0) (http://weblogo.threeplusone.com). The eSECIS core and the unpaired adenines (in either the apical loop of type I form or the bulge between the upper stem and the ministem of type II form) are highlighted in the box.

Among all predicted eSECIS-like structures, type I was the major form for *frhA*, *hdrA*, *frhD*, and *prxL* (gene encoding Prx-like protein) genes, whereas *selD* genes mainly have type II form. The nucleotide compositions of both SECIS core and apical loop regions of major and minor SECIS forms for different selenoprotein genes were examined (Fig. 4 and see online supplementary material for a colour version of Fig. S10). Although the nucleotides within the SECIS core of both type I and type II forms are mainly 5’-AUGA_GA-3′ which is consistent with previous observations [32–34], variations across different selenoprotein families and/or Asgard archaeal branches were observed, such as AUGA_AA in a number of *hdrA*/*frhD* and *frhA* genes in *Lokiarchaeia/Helarchaeales* and *Sifarchaeia/Borrarchaeales* and GUGA_GA in *selD* genes in *Thorarchaeia*. Similarly, despite that the first several nucleotides in the apical loop (type I) or in a small bulge at the top of the upper stem (type II) have been reported to be strongly conserved as adenines in both eukaryotes (AA) and *Lokiarchaeia* (AAAAA) [32], more variations were found in some other Asgard archaea, such as G[U/G]AAA in the majority of *frhA* genes in *Thorarchaeia* and *hdrA*/*frhD* genes in *Sifarchaeia/Borrarchaeales*, as well as [U/A][A/G]AA and AGAAA in most *selD* genes in *Thorarchaeia* and *Sifarchaeia/Borrarchaeales*, respectively. We also examined the nucleotide composition of sequences surrounding the key regions of eSECIS-like structures, including the internal loop and lower stem (Fig. 1A). Segment-based multiple alignments did not show highly conserved nucleotides among representative eSECIS-like structures in the five widespread selenoprotein families (See online supplementary material for a colour version of Fig. S11). However, family-specific nucleotide features could be observed for different selenoproteins. For example, [U/C]GG_C[C/U][A/G] and G[A/U]G_C[U/A]C base pairs were mostly detected in the lower stems of eSECIS-like structures of *frhA* and *selD* genes, respectively, which might be associated with Sec incorporation in the relevant selenoproteins. It should be admitted that current genomic sequencing data for the majority of Asgard archaea are still quite limited, resulting in the possibility that additional SECIS patterns have not been identified. Nonetheless, our results demonstrate more diverse features of eSECIS-like structures in Asgard archaea, which reflect a more complicated evolutionary scenario of SECIS elements in this archaeal group.

We also examined the presence of eaSECIS-like structures in Asgard archaea. Only a very small proportion of selenoprotein genes (49 sequences, 3.4%) were predicted to have such structures (Table S4). The majority of them (89.8%) co-exist with eSECIS-like structures (mostly downstream of eSECIS-like structures) in the 3’-UTRs of corresponding selenoprotein genes. Although these eaSECIS-like structures contain the conserved bulge-forming GAA_A nucleotides, no other nucleotides were found to be conserved across different selenoprotein genes (even in closely related species in the same clade, See online supplementary material for a colour version of Fig. S12). Moreover, almost all of them lack the consecutive S-S pairs in the upper stem which is highly conserved in the canonical eaSECIS elements of almost all selenoprotein genes in *Methanococcales* and *Methanopyrus* species. Given the rare occurrence and non-conserved nucleotide composition of these predicted eaSECIS-like structures and their co-localization with eSECIS-like structures, it is likely that they are not functional for Sec insertion in Asgard archaea. Further experimental studies are needed to verify this assumption.

With regard to the two newly identified selenoprotein families, only eSECIS-like structures could be detected in their selenoprotein genes (Table S5). Almost all of these structures are similar to those identified in the widespread selenoprotein genes (such as *frhA*, *hdrA*, and *frhD*) in same or closely related organisms. This implies that evolution of new selenoprotein genes from their Cys-containing homologs in Asgard archaea is a plausible route by acquisition of the SECIS elements from already existing selenoprotein genes, which may help to spread the Sec utilization trait vertically or laterally in this superphylum.

It was previously reported that the eaSECIS elements of *hdrA* and *frhD* genes in *Methanococcales* and *Methanopyrus* also have the AUGA_[G/A]A motif, which were proposed as the archetype for SECIS elements in *Lokiarchaeia*, and even eukaryotes [32]. However, it is unclear whether the same features are present in the eaSECIS elements of other selenoprotein genes (such as *frhA* and *selD*) in *Euryarchaeota*. Here, we carried out an extensive analysis of the eaSECIS elements of all selenoprotein genes in a variety of *Methanococcales* and *Methanopyrus* species (Table S6). Although no complete eSECIS-like structures could be found in the 3’-UTRs of their selenoprotein genes, all eaSECIS elements of *hdrA* and *frhD* genes detected in the two clades have the [A/G]UGA_[G/A]A motif (See online supplementary material for a colour version of Fig. S12, details in Table S6). Moreover, in *Methanopyrus*, the eaSECIS elements of all *frhA* and *selD* genes possess the AUGA_GA and GUGA_[G/A]A motifs, respectively. Such motifs could also be detected in the eaSECIS elements of *frhA* and *selD* genes in a small number of *Methanococcales* species, such as *Methanotorris igneus* Kol 5, *M. formicicus* Mc-S-70, and *Methanothermococcus thermolithotrophicus* DSM 2095 (*frhA*) and *Methanococcus maripaludis* C5 and *M. infernus* ME (*selD*). We could not find such features in the eaSECIS-like structures in Asgard archaea and in other selenoprotein genes (such as formate dehydrogenase alpha subunit (FdhA)) in *Methanococcales* and *Methanopyrus*. Thus, it seems that the [A/G]UGA_[G/A]A motif is more likely the archetype of eSECIS core, which has evolved in the SECIS elements of multiple selenoprotein genes in ancient archaea.

### Horizontal gene transfer of selenoprotein genes between Asgard archaea and *Euryarchaeota*

Horizontal gene transfer (HGT) events have been previously reported between Asgard archaea and *Euryarchaeota*, which are two evolutionarily disparate clades but may live in similar conditions or in symbiosis (such as certain *Lokiarchaeia* and euryarchaeal methanogens) [59, 60]. Considering that the majority of selenoprotein genes detected in Asgard archaea (including *frhA*, *hdrA*, *frhD*, and *selD* genes) are also present in *Methanococcales* and *Methanopyrus* (two relatively closely related orders in the *Methanomada* group from *Euryarchaeota*) and that *frhA*, *hdrA*, and *frhD* genes are often organized in the same operon in *Methanococcales* species [61], we tried to examine if HGT might occur between them (most likely from Asgard archaea to the common ancestor of *Methanococcales* and *Methanopyrus*).

**Figure 5:**
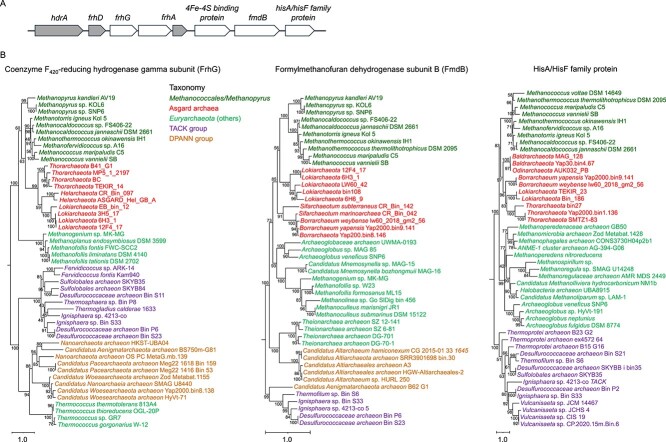
**Phylogenetic analyses of genes in or very close to the operon containing *hdrA*, *frhD*, and *frhA* genes.** (A) Organization of these genes in the majority of *Methanococcales* species. Selenoprotein genes are shaded; (B) phylogenetic analyses of different protein families. A maximum-likelihood phylogenetic tree was inferred for each family with the LG+C60+F+R model and 1000 ultrafast bootstrap pseudoreplicates. Each of the trees includes ~50 orthologous sequences from diverse species from different archaeal supergroups (Asgard archaea, *Euryarchaeota*, TACK, and DPANN groups). Names of species are color-coded according to different taxonomic classification. Paralogous sequences were used to root these trees. Bootstrap support values are indicated. The scale bar is shown at the bottom left of each tree.

Phylogenetic analyses of these selenoprotein genes might not provide informative results for distinguishing between HGT and vertical inheritance because they are only present in Asgard archaea and *Methanococcales*/*Methanopyrus* species. However, when HGT of selenoprotein genes occurred, genes surrounding them in the donor genomes might have also been co-transferred to recipient genomes. Thus, identification and phylogenetic analyses of these neighboring genes may provide evidence for the presence of HGT events. By examining genes within the genomic regions of 5 kb upstream and downstream of each of these selenoprotein genes in *Methanococcales* and *Methanopyrus* species and their distribution in different archaeal taxa, three most frequently detected genes were selected, namely coenzyme F_420_-reducing hydrogenase gamma subunit (FrhG), formylmethanofuran dehydrogenase subunit B (FmdB), and HisA/HisF family protein (Fig. 5A). Orthologous sequences of these genes from diverse species from different archaeal supergroups (Asgard archaea, *Euryarchaeota*, TACK, and DPANN groups) were identified using the strategy described in Materials and Methods. Maximum likelihood-based phylogenetic analyses of these genes in a variety of archaea reveal apparent HGT events between Asgard archaea and *Methanococcales*/*Methanopyrus* (Fig. 5B), which may support the hypothesis that the genomic fragment containing *hdrA*, *frhD*, *frhA*, and several other genes could have been transferred between Asgard archaea (potential donor) and the common ancestor of *Methanococcales* and *Methanopyrus* species (potential recipient). FmdB is a selenoprotein in *Methanococcales* and *Methanopyrus* species but a Cys-containing protein in Asgard and many other archaea. It is possible that Sec-containing FmdB evolved from a Cys-containing progenitor (derived from Asgard archaea) in the ancestor of *Methanococcales* and *Methanopyrus*. However, we could not detect significant HGT events for genes surrounding *selD*, implying that genomic contexts of *selD* genes have been disrupted in these organisms (See online supplementary material for a colour version of Fig. S13A). We also examined several most frequently observed neighboring genes of other Sec machinery genes (i.e., *secS*, *efSec*, and *pstk*) in *Methanococcales* and *Methanopyrus* genomes (See online supplementary material for a colour version of Fig. S13B-D). All of the examined genes are not located in close vicinity of Sec machinery genes in Asgard archaea. Although we could not find strong evidence for HGTs of most of these genes between Asgard archaea and these methanogenic archaea, a potential HGT event between *Methanococcales* and several Asgard archaea was observed for phosphoglycerate mutase (one of the neighboring genes of *secS*) (See online supplementary material for a colour version of Fig. S13B), suggesting a possibility of lateral transfer of the Sec pathway between the two evolutionarily distant clades. More studies are needed to verify if *Methanococcales/Methanopyrus* species acquired both Sec encoding system and selenoproteins from Asgard archaea. Anyhow, HGT appears to have played an important role in the evolution of Sec utilization trait in archaea.

## Discussion

It has been known for a long time that Se plays a vital role in a variety of organisms including bacteria, archaea, algae, fungi, and animals via participating in selenoprotein synthesis [2, 62, 63]. In the past two decades, much effort has been devoted to characterizing Sec encoding machinery and the categories and function of selenoproteins in both prokaryotes and eukaryotes [7, 16, 20–24, 44, 64–66]. Although biosynthesis of Sec and its insertion into proteins are similar between archaea and eukaryotes, their SECIS elements are completely different, resulting in an incomplete understanding of the origin of eukaryotic Sec incorporation machinery and its relationship with archaeal counterparts. Recent identification of the Sec pathway and eSECIS-like structures in several *Lokiarchaeia*, *Thorarchaeia*, and *Jordarchaeia* species provides preliminary clues for the existence of SECIS element intermediate form in these organisms related to eukaryotic common ancestor [32–34]. Here, we investigated the occurrence of Sec encoding machinery and selenoproteins in different branches of Asgard archaea based on a significantly increased number of Asgard archaeal MAGs. To our knowledge, these data represent the largest and most comprehensive view of Sec utilization in the Asgard superphylum.

Comparative analysis of Sec utilization trait reveals that this nonstandard amino acid could be used by the majority of Asgard archaeal species examined in this study, while parallel loss of this trait occurred in some clades. Further investigation of the selenoproteomes has generated the largest data set of selenoproteins in Asgard archaea, which not only extends current understanding of archaeal selenoprotein families but also helps to discover new selenoprotein-rich phyla and organisms. In the future, it would be valuable to identify new selenoproteins and to explore the roles and evolutionary dynamics of different selenoprotein families in Asgard archaea.

A significant contribution of the work is to develop generalized SECIS element models to predict eSECIS- and eaSECIS-like structures in Asgard archaeal selenoprotein genes. The majority of Asgard archaeal selenoprotein genes possess eSECIS-like sequence and structural features, implying that eSECIS-related Sec insertion mechanism has been widely adopted for selenoprotein biosynthesis in this archaeal group closest to eukaryotes. Although type I eSECIS-like structure was the dominant form for almost all selenoprotein genes detected here, most *selD* genes possess type II form. This is consistent with previous observations that almost all SECIS elements of eukaryotic *selD/sephs2* genes are of type II [67], indicating that the eSECIS element of *selD*/*sephs2* gene is directly derived from the eSECIS-like structure of Asgard archaeal *selD* genes. Compared to highly conserved characteristics in eSECIS elements, varying degrees of diversity in key regions of eSECIS-like structures (i.e. SECIS core and first several nucleotides in the apical loop) are present in different selenoproteins and branches of Asgard archaea. Moreover, we found same or very similar eSECIS-like motifs in the eaSECIS elements of multiple selenoprotein genes in *Methanococcales* and *Methanopyrus* species, implying that the archetype of eSECIS element (especially the eSECIS core) has already been present in ancestral archaeal selenoprotein genes. Although eaSECIS-like structures were observed in a small number of Asgard archaeal selenoprotein genes, they are probably unrelated to Sec incorporation.

**Figure 6:**
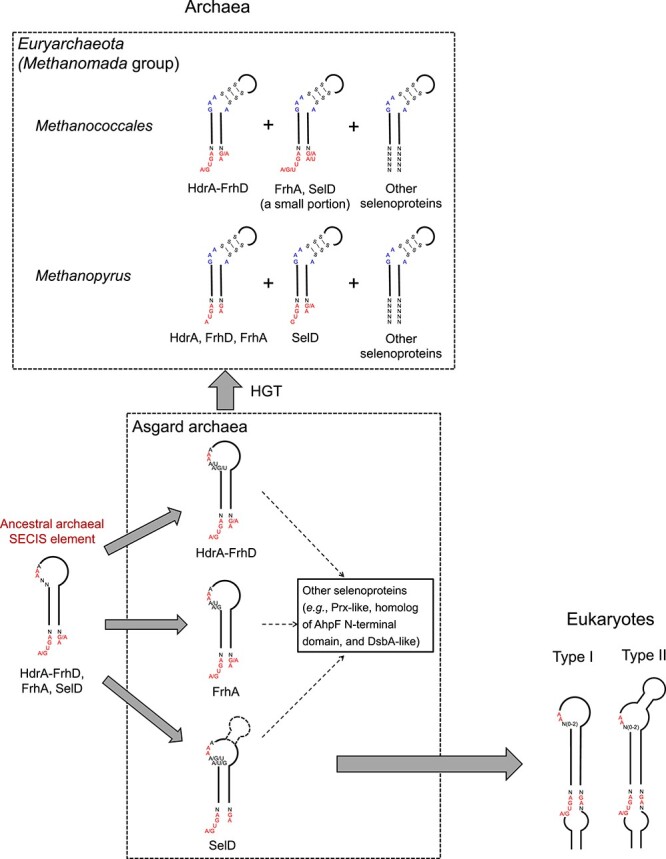
**A proposed roadmap for the transition of SECIS element from archaea to eukaryotes.** Conserved features present in eSECIS elements and the GAA_A bulge conserved in eaSECIS elements are shown in different colors. HGT, horizontal gene transfer.

Given the remarkable similarity and diversity of eSECIS-like structures in Asgard archaea and possible HGTs of selenoprotein genes between Asgard archaea and *Euryarchaeota*, a possible roadmap for the evolution of archaeal SECIS element and its transition to eukaryotes is proposed, which provides a more comprehensive and reasonable explanation for the evolutionary scenario of this key *cis*-acting RNA structure involved in Sec incorporation into selenoproteins (Fig. 6). The archetype of eSECIS core ([A/G]UGA_[G/A]A) and the unpaired AA in the apical loop region might have existed in ancestral archaeal SECIS elements of several selenoprotein genes (e.g. *hdrA*, *frhD*, *frhA*, and *selD*) in the ancestor of Asgard archaea, and was then preserved in multiple clades of this superphylum. The GA_GA quartet has been known as a typical motif in various kink-turn structures, and the GA_AA pairing was also previously reported to fold into a potential kinked geometry in certain RNAs, such as the small ribosomal subunit and the thiamine pyrophosphate riboswitch in some microbes [68, 69]. Among all identified Asgard archaeal selenoproteins, SelD is the only one whose members are also widespread in eukaryotes. Considering that SelD is a key component of the Sec encoding system, additional selenoproteins were presumably present in the last common ancestor of eukaryotes. Although the composition of the ancestral eukaryotic selenoproteome is still unclear, the ancestor of extant eSECIS elements was at least partially derived from the eSECIS-like structures in *selD* gene (mainly type II). Thus, SelD may serve as an intermediate for the archaea-to-eukaryote transition of Sec insertion machinery besides its roles in Sec biosynthesis and maintaining the Sec encoding trait as a selenoprotein. Moreover, the eSECIS-like structures in *selD* possess more eukaryotic features. For example, a significant number of eSECIS-like structures in *selD* genes in *Thorarchaeia* contain the GUGA_GA motif in the SECIS core, which is also present in many selenoprotein genes in several green alga (e.g. *Ostreococcus tauri* and *O. lucimarinus*) [70]. On the other hand, *Methanococcales* and *Methanopyrus* species acquired these selenoprotein genes and eSECIS-like structures from Asgard archaea via HGT events. In these methanogenic archaea, the [A/G]UGA_[G/A]A motif might become nonessential for Sec incorporation whereas new features (such as the GAA_A bulge and consecutive S-S pairs in the upper stem) have evolved to form the eaSECIS element. Additional selenoproteins (such as FdhA and FmdB) then evolved by acquisition of the eaSECIS elements from existing selenoprotein genes. Although there is no strong evidence that *Methanococcales*/*Methanopyrus* species acquired Sec encoding system from Asgard archaea via HGTs, lateral acquisition of Sec machinery genes is possible due to the potential presence of HGT events between the two clades for certain genes surrounding them. Considering the bias of distribution of sequenced archaeal genomes, it is still unclear whether the Sec utilization trait and ancestral archaeal SECIS element appeared preceding the split of Asgard archaea and *Euryarchaeota* or other archaeal clades and whether other archaea can use this nonstandard amino acid. Further studies are needed to confirm our hypothesis and to elucidate more mechanistic and evolutionary details of archaea-to-eukaryote transition of Sec machinery with the increasing of new archaeal genomes.

Although we did not find significant homologs of SBP2 (an essential factor in eukaryotic selenoprotein synthesis) in Asgard archaea, the possibility that unknown genes are needed to perform similar function could not be excluded. In eukaryotes, SECIS binding from SBP2 recruits EFSec protein, leading to the recoding of the UGA codon and Sec incorporation. Multiple alignment of archaeal EFSec proteins reveals that EFSec sequences from Asgard archaea contain specific residues (two conserved glycines) that might be related to Sec incorporation in this archaeal group (See online supplementary material for a colour version of Fig. S14). In addition, by analyzing the gene neighborhood for different Sec encoding components in Asgard archaeal MAGs, a hypothetical protein with unknown function (e.g. MBD3157761.1) was found to be potentially associated with the Sec pathway, whose genes were exclusively present in this superphylum and were often located close to Sec machinery genes such as *secS*, *efSec*, and *pstk*. A future challenge would be to verify these findings and to discover new mechanisms and evolutionary trends related to Sec synthesis and incorporation in Asgard archaea.

In conclusion, we have carried out a comprehensive comparative analysis of Sec encoding machinery in hundreds of Asgard archaeal species and produced the largest map of Sec utilization in this evolutionarily important archaeal superphylum. Our data suggest that Sec utilization was an essential trait for the ancestor of Asgard archaea. Identification of selenoproteomes and selenoprotein-rich phyla and species may extend current knowledge on the diversity and evolution of selenoproteins in archaea. Further investigation of eSECIS-like structures in different selenoprotein genes reveals a complex and dynamic evolutionary process of SECIS element in archaea. HGT might be an important mechanism in the evolution of eaSECIS element in euryarchaea. Lastly, a roadmap for the transition of SECIS element from archaea to eukaryotes was proposed, and SelD may play a role in this process.

## Supplementary Material

Supplementary_data_2_wrae111

## Data Availability

All genomic assemblies used are available through NCBI accession numbers indicated in Table S3. All selenoprotein sequences in Asgard archaea identified in this work are available in Supplementary data 1. Raw data and results for multiple sequence alignments and phylogenetic analyses of different selenoprotein families, genes in or very close to the *hdrA*-*frhD-frhA* operon, and genes surrounding Sec machinery genes are available in Supplementary data 2.
